# Peripartum Cardiomyopathy Presenting With Severe Biventricular Dysfunction, Valvular Regurgitation, and Familial Predisposition: A Case Report

**DOI:** 10.1002/ccr3.72829

**Published:** 2026-05-31

**Authors:** Muhammad Ibrahim Shah, Hasnain Wajeeh Saqib, Maaz Khan, Muhammad Saeed Ullah Shah, Dadullah Faiz

**Affiliations:** ^1^ Shifa College of Medicine Islamabad, Rawalpindi Pakistan; ^2^ Riphah International University Islamic International Medical College Islamabad, Rawalpindi Pakistan; ^3^ Shifa International Hospital Islamabad, Rawalpindi Pakistan; ^4^ Faculty of Medicine Kandahar University Kandahar Afghanistan

**Keywords:** heart failunre, medical therapy, peripartum cardiomyopathy, pregnancy complication

## Abstract

Peripartum cardiomyopathy (PPCM) is defined as the onset of left ventricular systolic dysfunction either during pregnancy or up to 5 months after delivery, without any other known cause of heart failure. The exact pathogenesis of this disease is unknown, and its prognosis remains poorly understood. We present a case of a 27‐year‐old woman presenting at 37 + 3 weeks of gestation, with reduced ejection fraction, biventricular systolic dysfunction and dilation, and valvular regurgitation. She was managed with medications slightly different from guideline‐mediated standard therapy. The unique and most crucial detail of this case is that it showed a close family history of PPCM, as the patient's older sister had also died of the same cause. Learning objectives: genetic and familial risk of developing peripartum cardiomyopathy.

## Key Clinical Message

A 27‐year‐old woman at 37 weeks of gestation presented with biventricular heart failure and severe systolic dysfunction. Notably, the patient's older sister previously died from peripartum cardiomyopathy. This rare familial occurrence suggests a potential genetic predisposition and highlights the need for vigilant screening in siblings of affected patients to improve outcomes.

## 1 Introduction

Peripartum cardiomyopathy (PPCM) is a rare, life‐threatening form of left ventricular systolic dysfunction that develops during the last month of pregnancy or within 5 months after delivery, without any other identifiable cause of cardiomyopathy [1]. The condition is classically characterized by left ventricular systolic dysfunction, although disease severity and clinical outcomes vary widely. The global incidence of PPCM is estimated at 1 in 1000–4000 live births, but higher rates may be observed in certain geographic regions [2].

Although the exact pathogenesis is unknown, it is hypothesized that unbalanced peripartum or postpartum oxidative stress may trigger the proteolytic cleavage of the hormone, prolactin, into a proapoptotic, antiangiogenic, and proinflammatory fragment [3]. Another hypothesis suggests that disruption of cardiac angiogenesis, mediated by hormonal and immune changes during pregnancy, may also contribute to the development of PPCM [4]. Recent studies also suggest a role of truncating variants in the TTN gene, which encodes a sarcomere protein called titin, and other cardiomyopathy‐related mutations in the familial clustering of PPCM, suggesting a genetic interplay [5, 6].

Here, we describe the case of a young woman with PPCM who had significant biventricular dysfunction with valvular regurgitation. There is also a noteworthy family history of an elder sibling who died from postpartum cardiac failure. The significance of early detection, family risk assessment, and long‐term care is highlighted by this instance.

## 2 Case Report

A 27‐year‐old woman, gravida 4 para 2 + 1, presented at 37 + 3 weeks of gestation with complaints of shortness of breath on exertion and orthopnea. She had no prior cardiovascular disease, hypertension, or diabetes. Her obstetric history included one miscarriage and two live births.

Notably, her elder sister, married to the same husband, had developed severe heart failure within a month after her delivery, with an echocardiographic ejection fraction (EF) of 15%, and died within 1 month despite medical management. Her father had also died due to a heart attack.

### 2.1 Investigations

**FIGURE 1:**
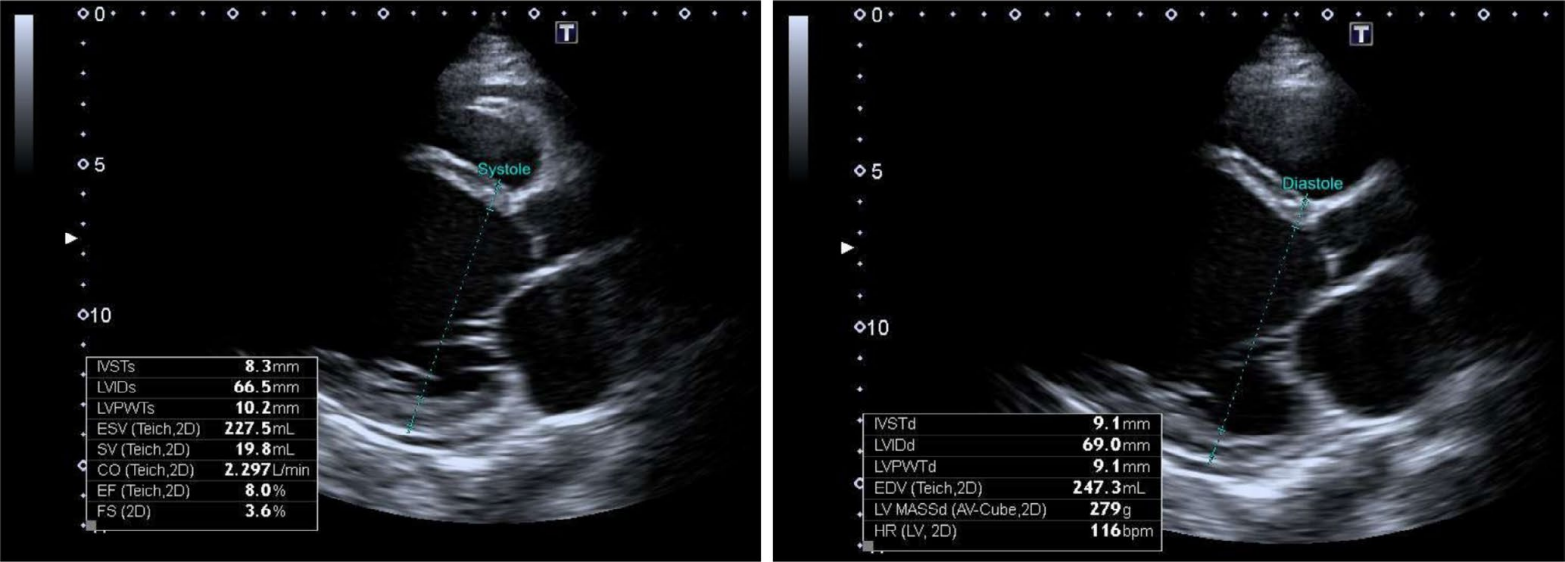
Echocardiogram showing increased left ventricular diameter during systole and diastole.

**FIGURE 2:**
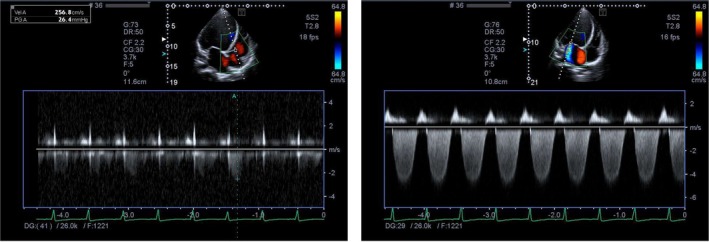
Pulsed‐wave Doppler and color and continuous‐wave Doppler showing regurgitant flow across the atrioventricular valves, consistent with moderate‐to‐severe functional mitral and tricuspid regurgitation secondary to ventricular dilation.

**FIGURE 3:**
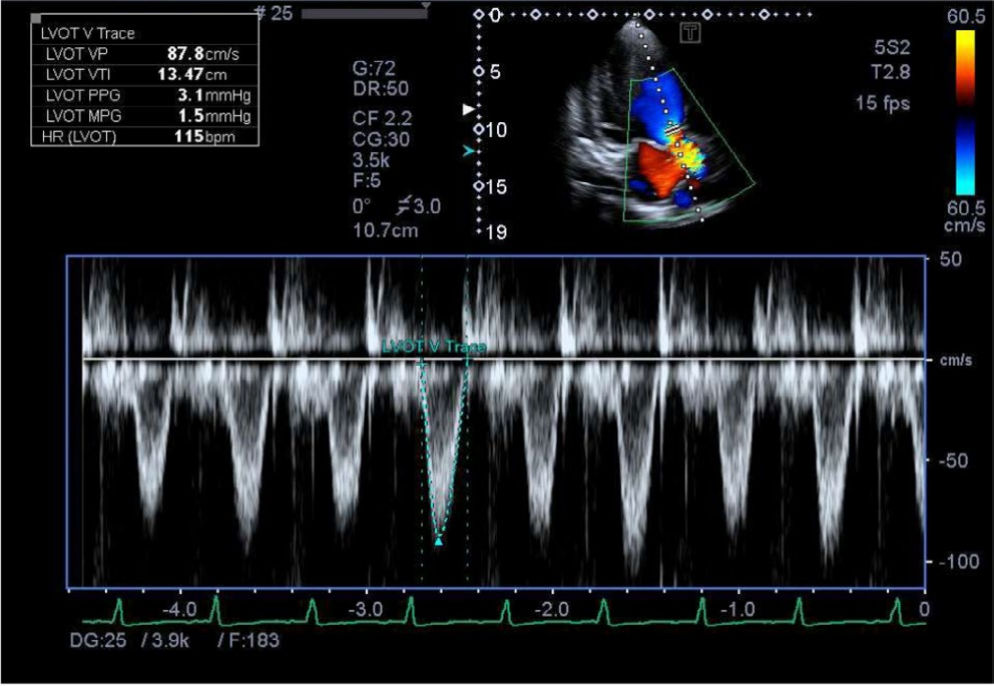
Doppler echocardiogram showing left ventricular systolic dysfunction.

- Electrocardiogram: Normal sinus rhythm.
- Echocardiogram: Ejection fraction of 25%. Left ventricular end diastolic diameter of 69. Figure 1 severely dilated left ventricle. Severely dilated left atrium. Moderate‐to‐severe mitral and tricuspid regurgitation. Figure 2 Poor left ventricular systolic function. Moderate‐to‐severe right ventricular systolic dysfunction. Figure 3.
- Ultrasound abdomen: Mild ascites. Bilateral moderate pleural effusion. Borderline hepatomegaly.
- CT pulmonary angiography: No pulmonary embolism or DVT. Moderate right and mild left pleural effusion with consolidation in the lungs' lower lobes, patchy ground glass and areas of airspace opacification in both lungs; interlobular smooth thickening with congested hila.
- Gynecological evaluation: Mild‐to‐moderate pelvic ascites and an enlarged uterus

A diagnosis of PPCM was established based on timing, echocardiographic findings, and exclusion of other secondary causes.

The patient was started on diuretics and underwent emergency cesarean section along with bilateral tubal ligation under spinal anesthesia, delivering an infant who required neonatal intensive care unit (NICU) admission. She was shifted to the surgical step‐down for a day for further monitoring, and cardiology was taken on board to optimize her medications. One day later, she was moved to the cardiac care unit, where she was given intravenous furosemide, and her symptoms improved.

### 2.2 Follow‐Up

The patient started breathing comfortably on room air. She had sinus tachycardia, which was managed with medications. She was prescribed clopidogrel and aspirin (75 mg), furosemide (40 mg), metoprolol tartrate (25 mg), spironolactone (25 mg), losartan potassium (25 mg), and digoxin (0.25 mg), all administered as oral tablets. The patient had gotten stable, with a New York Heart Association (NYHA) Grade 1, and was discharged home. Her child was put on formula milk as the patient avoided breastfeeding.

## 3 Discussion

PPCM can be a distinct clinical entity with a variable prognosis, ranging from complete recovery to refractory heart failure and even death [7]. The majority of the PPCM patients present with isolated left ventricular dysfunction; however, our patient developed severe biventricular dysfunction with valvular regurgitation, a rarer presentation associated with worse outcomes [8]. The immediate family history also highlights the clinical significance of our case, as it was the first observed case of PPCM with a family history.

### 3.1 Possible Prognostic Implications

Initial EF is a key predictor of recovery. Women with EF < 30% at diagnosis have a significantly lower likelihood of regaining normal function. Subsequent pregnancy may also lead to worse outcomes and even cause death [9].

### 3.2 Familial and Genetic Predisposition

The death of the patient's sister from identical postpartum cardiac dysfunction suggests a familial component. Recent genetic studies demonstrate that 15%–20% of patients with PPCM harbor pathogenic variants in cardiomyopathy genes, particularly TTN truncating variants [5]. Familial clustering has been reported in case series, underscoring the need for genetic counseling and cascade screening [4, 6]. Screening in asymptomatic pregnant women with an immediate family history of PPCM could catch the disease early in its progression and help prompt management.

### 3.3 Management Challenges

Recent guidelines from the European Society of Cardiology (ESC) recommend the use of HFrEF pharmacological therapy in all patients, while respecting pregnancy‐related contraindications. Blockade of prolactin release using bromocriptine as a disease‐specific therapy in addition to standard heart failure treatment has also shown promising results in recent clinical trials. The importance of education and counseling around contraception and future pregnancies is also highlighted [10].

### 3.4 Clinical Implications

This case raises important considerations:

1. Familial risk: Screening relatives of affected women may enable early detection.
2. Follow‐up adherence: Early postpartum discharge must be balanced with structured monitoring.
3. Research need: Familial PPCM warrants further genetic and environmental exploration.

## 4 Conclusion

This case further supports the established association between PPCM and familial or genetic predisposition. Given the patient's family history and the emerging evidence linking pathogenic variants to PPCM, detailed family history assessment and consideration of genetic counseling remain important components of clinical evaluation.

## Author Contributions

**Muhammad Ibrahim Shah:** conceptualization, methodology, data curation, writing – original draft, investigation. **Muhammad Saeed Ullah Shah:** supervision, resources, project administration, validation, writing – review and editing. **Maaz Khan:** methodology, visualization, writing – review and editing, validation. **Dadullah Faiz:** conceptualization, supervision, writing – review and editing, methodology. **Hasnain Wajeeh Saqib:** investigation, visualization, writing – original draft, writing – review and editing.

## Funding

The authors have nothing to report.

## Ethics Statement

Ethics grant was obtained by the IRB of Shifa International Hospital.

## Consent

Written consent was obtained from the patient.

## Conflicts of Interest

The authors declare no conflicts of interest.

## Data Availability

The data supporting the findings of this case report are available within the article. No additional datasets were generated or analyzed during the current study. Further details may be available from the corresponding author upon reasonable request, subject to patient confidentiality and institutional regulations.
